# The integration of emotional and symbolic components in multimodal communication

**DOI:** 10.3389/fpsyg.2015.00961

**Published:** 2015-07-07

**Authors:** Marc Mehu

**Affiliations:** Department of Psychology, Webster Vienna Private University, Vienna, Austria

**Keywords:** emotional communication, multimodal communication, social signals, ethology, non-verbal communication, pragmatics

## Abstract

Human multimodal communication can be said to serve two main purposes: information transfer and social influence. In this paper, I argue that different components of multimodal signals play different roles in the processes of information transfer and social influence. Although the symbolic components of communication (e.g., verbal and denotative signals) are well suited to transfer conceptual information, emotional components (e.g., non-verbal signals that are difficult to manipulate voluntarily) likely take a function that is closer to social influence. I suggest that emotion should be considered a property of communicative signals, rather than an entity that is transferred as content by non-verbal signals. In this view, the effect of emotional processes on communication serve to change the quality of social signals to make them more efficient at producing responses in perceivers, whereas symbolic components increase the signals’ efficiency at interacting with the cognitive processes dedicated to the assessment of relevance. The interaction between symbolic and emotional components will be discussed in relation to the need for perceivers to evaluate the reliability of multimodal signals.

## Introduction

This article revolves around two ideas that have stayed, in my opinion, on the fringes of research in human communication. The first idea is that the primary function of social signals is to influence perceivers, i.e., to produce responses in others that are beneficial to signalers. This idea has been discussed extensively in the field of animal behavior (Owren et al., 2010; Stegmann, 2013), but less so in human communication (for an exception, see Owren and Bachorowski, 2003). The second idea defended in this paper is that multimodal communication has emerged as the result of an interaction, over human evolutionary history, between signaler and perceiver roles (for a similar argument in animal communication research, see Guilford and Dawkins, 1991; Rowe, 1999). In particular, I will argue that different elements of multimodal signals have evolved to address the selective pressures presented by two cognitive strategies perceivers use to process and respond to signals: an evaluation of relevance and an assessment of reliability. The goal of this article is to call for an integration of information transfer and social influence accounts of communication in a coherent framework informed by evolutionary theory.

## Information Transfer and Social Influence in Human Communication

Social signals have been studied in many disciplines and the range of definitions for this concept is relatively broad (Mehu et al., 2012a). Borrowing the terminology developed by ethologists to define animal signals (see Maynard Smith and Harper, 2003), Mehu and Scherer (2012) have proposed a definition of human social signals that integrates the symbolic character of human communication with the more evolutionarily ancient properties of animal signals: “human social signals are acts or structures that influence the behavior or internal state of other individuals, that evolve because of that effect, and that are effective because the perceiver’s response has also evolved; signals may or may not convey conceptual information or meaning” (p. 399). In this framework, human social signals are tangible units of communication that can be perceived as visual, auditory, olfactory, or tactile stimuli. As such, the physical properties of social signals constitute raw information on the basis of which perceivers take social decisions. These decisions can be adaptive for the perceiver if the signal’s physical properties correlate with some psychobiological processes in which a perceiver has an interest (e.g., reproductive state, behavioral intentions, attitudes, cognitive evaluations, physiological reactions, subjective feelings, etc.). Although the material properties of social signals can be correlated with unobservable psychobiological processes, to conflate signals and their possible referents is inadequate as it does not help understand the complexity of communication. For example, it is hard to conceive that unobservable psychobiological processes are at the same time social signals and the referents of non-verbal communicative units. A more plausible assumption is that social signals are the means with which psychobiological processes like cognition, emotion, and attitudes are implemented in everyday social interactions.

Another aspect of social signals is their evolutionary function, i.e., how social signals increase survival and reproductive success of the individual who displays them. In the framework presented here, a signal’s function is to produce a response in the perceiver that is adaptive to the signaler. I argue that a signal fulfills its function in a number of ways, and the diversity of these ways results from an evolutionary process whereby perceivers (the main targets of signals) have placed selective pressures on signalers in order to maximize adaptation to the social environment and to avoid social exploitation. The pairing between conceptual information and physical properties of non-verbal behavior is one way social signals achieve their function of social influence. Therefore, information transfer is a way social signals fulfill their function of social influence rather than a separate function in itself (see also Scarantino, 2013).

The mainstream view on non-verbal communication posits that signalers encode information relative to internal states (emotion, cognition, attitudes, social motives, and dispositions) in a signal, and that the signal is decoded by perceivers who then retrieve the information. Although the inference of a signaler’s internal states is important for perceivers in most social situations, the faithful encoding of these states may not always be adaptive for signalers themselves because perceivers may act against a signalers’ goals (Grammer et al., 1997). In my opinion, the fact that it is so important for perceivers to form a reliable representation of the social environment has inflated the importance, in the eyes of psychologists, of the disclosure of unobservable psychobiological processes by signalers. There are many situations in which signalers have an advantage either in concealing information that could be used by the perceiver to act at the expense of the signaler, or in using deception. As a general rule, when there is a conflict of interest between signalers and perceivers, it is expected that the signaler will (a) retain valuable information, (b) try to influence perceivers to its own advantage, or (c) use deceptive signals (Maynard Smith and Harper, 2003). On the other hand, when a given interactive outcome is advantageous for both signalers and perceivers it is expected that reliable transfer of information will take place because none of the parties involved would benefit from deceiving the other. Krebs and Dawkins (1984) have argued that the nature of signals should depend on these contextual aspects. For example, when signaler and perceiver have conflicting interests, signals will tend to be more intense in order to be more effective in producing a response in perceivers; while signals will be less conspicuous when signalers and perceivers both benefit from reliable disclosure of internal states and behavioral intentions. Therefore, contextual factors determine whether it is adaptive for a signaler to accurately convey internal states, or to make strategic efforts to either conceal their intentions or use manipulation tactics. This implies that inferences made by perceivers will not only be based on the signal itself but also on how the signal interacts with situational cues.

A model of communication that is purely based on information transfer is unlikely to help us understand the complexity of communication (Wilson and Sperber, 2006; Owren et al., 2010; Scott-Phillips and Kirby, 2013). More specifically, such a model would fail to recognize that the roles of signaler and perceiver, although complementary, have different functions. On the one hand, signalers produce signals that have a high impact on perceivers and, on the other hand, perceptual systems optimize the use of information that can be gleaned from the situation in which communication takes place. This idea is based on Owings and Morton’s (1997) model of animal communication whereby communication is seen as a dynamic process that entails the management and assessment of the social environment by signalers and perceivers. In this model, information transfer is seen as secondary and is considered adaptive in only a fraction of the situations in which people communicate (Owren et al., 2010), namely when signalers and perceivers would both benefit from the reliable transfer of information. Therefore, depending on the situation, the communication process does not serve signalers and perceivers in the same way. Although in most cases the signaler should benefit from producing a desired response in the perceiver, the latter should mostly benefit from gaining adaptive social information. The signaler would only benefit from sending reliable signals when perceivers make their responses conditional on the acquisition of relevant and reliable information. It is the task of the researcher to determine whether the situation favors reliable transfer of information or social influence. This is likely to depend on the costs incurred by perceivers to respond to a signaler’s displays. Consequently, the interaction between information transfer and social influence will depend on the respective costs incurred by signalers and perceivers in a given context.

## The Complexity of Human Communication is Reflected in Multimodal Signals

The concept of multimodal communication follows the observation that social signals are complex and cover several sensory modalities (Johnstone, 1996; Partan and Marler, 1999; Rowe, 1999). Signals can also have multiple components within a particular modality. For example, visual signals entail motor components (movements produced by muscular activity), morphological components (structure and shape of particular body parts), or color components (e.g., skin or hair coloration). Within the framework of information transfer, multimodal signals have been proposed to function in two different ways (Johnstone, 1996): By redundantly encoding the same information in several channels, and by varying the nature of the information conveyed in the different channels. The first solution (backup signals) ensures that the message is transmitted, even when environmental circumstances prevent one of the channels to operate (e.g., in poor light conditions, or in noisy environments). The second solution (multiple messages) increases the amount of information transferred by using different channels to convey additional information. From a social influence perspective, multimodal signals could be more efficient at influencing perceivers because their complex structure makes them better at interacting with perceivers’ psychological mechanisms. Evidence from the field of animal communication suggests that multimodal signals are more easily detected, discriminated, and memorized (Rowe, 1999). In humans, the presentation of audio-visual signals appear to have a different impact on perceivers than the separate presentation of single modalities (Mehu and van der Maaten, 2014).

The present article defends the idea that the combination between different components or modalities of a signal has evolved to meet the requirement imposed by assessment systems. Perceivers’ social decisions have relied increasingly on mental inferences involving the interaction between multiple indicators (mostly cues and signals emitted by signalers as well as situational features). Such inferences could function to resist social exploitation and manipulation by signalers and to optimize social decision making. Increasing cognitive complexity in primates (Dunbar and Shultz, 2007) placed a selective pressure on signals to become more efficient at interacting with perceiver’s filtering mechanisms. On the other hand, evolutionary in-built robustness in primate signals could be a fertile bed for the evolution of more complex signals, which components could take on new functions in communication (Ay et al., 2007). I consider the transfer of abstract and conceptual information as one of these new functions, which is fulfilled by the symbolic components of multimodal signals. The symbolic components cut across the visual and auditory modalities (visual symbols and speech are two examples of these components in two different modalities) and their form is relatively arbitrary with regards to their function, which is to interact with representational structures of the mind. The evolution of the symbolic component of human communication has paralleled the development of voluntary motoric capabilities necessary for the production of communicative units at the acoustic (speech) but also the visual level (e.g., gestures). The increased voluntary control over this component facilitated signal production and the expression of intentions, but it also created a new opportunity for signalers to take advantage of perceivers’ assessment systems and produce potentially deceptive signals.

The integration of symbolic components in communication enhances the efficiency of signals at producing desired responses in perceivers because such components allow signalers to provide information which perceivers have an interest in. By using symbolic components, signalers can also clarify the type of response sought in perceivers. By bringing elements that are absent in the current situation but nonetheless relevant to it, the symbolic component of a signal also broadens the communication context and the perceivers’ opportunities to pose adaptive actions. Symbolic communication is therefore adaptive for signalers because it helps them influence perceivers more efficiently. Due to the increased potential it offers for assessment of the physical and social environments, this mode of communication is likely to have been selected by perceivers during evolutionary history. The increased voluntary control over signal production (in particular over the symbolic components) allows more flexibility in communication and a better relationship with cognitive executive functions such as memory and planning. It also gives more opportunities for signalers to deceive perceivers by sending false information. This created a selective pressure on perceivers to develop resistance mechanisms designed to evaluate the reliability of the source. It has been argued that, in addition to the evaluation of an utterance’s relevance, humans have developed cognitive mechanisms to evaluate a signaler’s reliability (Sperber et al., 2010). I argue that when evaluating the trustworthiness of a signal, perceivers use other cues or indices present in the signal that are difficult to manipulate or control voluntarily, for example emotional components.

Emotional expressions have been found to strongly influence person perception (Knutson, 1996; Hess et al., 2000). In line with the idea that emotions are essential to maintain commitment to social contracts (Hirshleifer, 1987; Frank, 1988) it was found that emotional expressions could function as reliable indicators of behavioral intentions and interpersonal dispositions such as prosociality (Brown et al., 2003; Mehu et al., 2007) or threat (Reed et al., 2014). The reason why perceivers would rely on emotional cues to make adaptive social decisions is that these cues reflect automatic psychobiological processes that are responsible for the production of adaptive behavior that may also have implications for the perceiver’s adaptation. Therefore, the initial stages of emotion-based behavioral sequences are informative as they allow to predict future behavior and anticipate adjustment to a situation. It is therefore adaptive for perceivers to react emotionally to a range of observed emotional cues, and to consider these cues as important sources of social information (van Kleef, 2009). Inferences about the behavioral intentions of signalers is an important goal for perceivers and the accuracy of these inferences likely determines whether reactions to emotional displays of others are adaptive in the long run. Facial signs of enjoyment displayed in parallel to verbally expressed intentions to cooperate can be predictive of cooperative moves (Reed et al., 2012), suggesting that emotional signals could be used to evaluate the reliability of verbal claims. Therefore, when evaluating multimodal signals that contain symbolic components, perceivers could give particular attention to the emotional components of the signals as the latter may ensure the reliability of the former (Mehu and Scherer, 2012).

Emotional signals lead to emotional reactions in perceivers (Forgas, 1998; Owren and Bachorowski, 2003; van Kleef, 2009), and these emotional reactions can modify the perceiver’s thoughts and behavior to the advantage of the signaler (van Kleef et al., 2004). I argue that emotionality is a property of multimodal communication that makes it more efficient at producing responses in perceivers that are adaptive to signalers. In this view, emotion does not represent the content of a signal that is encoded by a signaler in order to be decoded by a perceiver, but one of a signal’s properties, which is activated by a series of automatic cognitive and physiological processes that are difficult to control voluntarily. There are two ways emotional processes make a signal more efficient. The first is by modifying the physical properties of the signal and making it more intense, more salient, and more variable, hence more difficult for perceivers to resist to, to ignore, or to habituate to. Second, emotional processes make a signal more efficient by acting on the cognitive processes which function is to evaluate a signal’s authenticity. In support to this view, perceived authenticity of an expression is related to the intensity of the facial cues that are more difficult to control voluntarily (Mehu et al., 2012b). In this context, emotional authenticity can be conceived as the likelihood that the signal is associated with the cognitive, physiological, and experiential processes involved in the coordination of adaptive responses (Scherer, 2005). In other words, an emotionally authentic signal is a good predictor of the signaler’s tendency to react in a particular situation. In day-to-day communication, emotional signals act in parallel to symbolic signals to make the overall multimodal signal appear more salient in the eyes (or ears) of perceivers and to make the information content of the symbolic component more reliable. Rather than to transfer information about emotional states, the function of emotional signals is therefore to optimize the effect of multimodal signals on perceivers.

## Conclusion

The question of what is transmitted in non-verbal communication has kept researchers busy for the last decades. Looking for information about emotion or its components (Ekman et al., 1980; Scherer and Grandjean, 2008), information about social motives (Fridlund, 1994; Parkinson, 2005), information about personality (Hall et al., 2005), or information about attitudes (Mehrabian, 1971), non-verbal communication research has been on an incessant quest for signal meaning. In my opinion, the strong focus on questions of meaning is based on excessive reliance on the view that communication mostly functions to transfer information. Models of information transfer are useful to understand certain aspects of symbolic communication, but they have to be complemented with models that emphasize social influence. Such integration implies that we recognize the different functions associated with the roles of signaler and perceiver in communication. Although these two roles interact to a great extent and have co-evolved during human evolutionary history, one cannot necessarily assume that signalers’ goals are to serve perceivers’ goals. With this in mind, research should pursue questions related to what is achieved by communicative signals and by perceivers’ assessment mechanisms, along with a careful analysis of the contextual factors and interactive consequences of multimodal displays.

I propose that multimodal signals that include both symbolic and emotional components are advantageous for signalers in that they are more likely to produce the adequate response in perceivers because (a) they contain information necessary for perceivers to evaluate the signal in relation to context (they target perceiver’s evaluations of relevance) and (b) they show appropriate correlation with social information adaptive to perceivers (they target perceivers’ evaluation of the trustworthiness of the source). Future research needs to clarify the processes involved in the production of multimodal signals (for example the appraisal processes underlying emotional communication, Mortillaro et al., 2013) as well as the role of abstract, language-based, representational structures as possible meditators of perceivers’ responses to signals. Finally, investigating the costs and benefits for signaler and perceiver that are inherent to the context in which communication takes place should also constitute an important element of future study designs in social signal processing research.

### Conflict of Interest Statement

The author declares that the research was conducted in the absence of any commercial or financial relationships that could be construed as a potential conflict of interest.
